# Progressive Medicine. Fifth Annual Series. Volume II, June, 1903

**Published:** 1903-10

**Authors:** 


PROGRESSIVE MEDICINE. FIFTH ANNUAL SERIES. VOLUME II, June, 1903.—A Quarterly Digest of Advances, Discoveries and Improvements in the Medical and Surgical Sciences. Edited by Hobart Amory Hare, M. D., Professor of Therapeutics and Materia Medica in the Jefferson Medical College of Philadelphia. Octavo, handsomely bound in cloth, 427 pages, with 46 illustrations. Per volume, $2.50, by express prepaid. Per annum, in four cloth-bound volumes, $10.00. Lea Brothers & Co., Publishers, Philadelphia and New York.

This number of *Progressive Medicine* contains a timely article on "The Surgery of the Abdomen, Including Abdominal Hernia," by Dr. Wm. B. Coley, a well written section on "Gynecology," treating chiefly the various phases of cancer of the uterus, by Dr. John G. Clark; a section on "Diseases of the Blood," by Dr. Alfred Stengel; and a section on Ophthalmology, edited by Dr. Edward Jackson. The illustrations are numerous and excellent. R. & L.

